# Thyroid Dysfunction and Cognitive Impairment: Neural Mechanisms and Neurofeedback‐Based Interventions

**DOI:** 10.1002/brb3.71817

**Published:** 2026-09-30

**Authors:** Zhiqiang Ma, Yutong Liu, Mengyuan Chen, Mengyang Wang, Tao Liu

**Affiliations:** ^1^ Department of Breast and Thyroid Surgery (Second Ward) The First Affiliated Hospital of Henan University Kaifeng Henan Province China; ^2^ School of Management Shanghai University Baoshan Shanghai China; ^3^ School of Health Fujian Medical University Fuzhou Fujian China

**Keywords:** cognitive impairment, neural mechanism, neurofeedback, thyroid dysfunction

## Abstract

**Background:**

Thyroid dysfunction has emerged as a significant contributor to global public health challenges, with mounting evidence indicating its impact on cognitive function.

**Methods:**

The current literature was synthesized to examine the association between thyroid dysfunction and cognitive impairments, the underlying neural mechanisms, and potential therapeutic approaches.

**Results:**

A critical gap identified in this review is the incomplete understanding of the neural mechanisms underlying thyroid‐related cognitive decline, which hampers the development of targeted interventions.

**Conclusion:**

Neurofeedback is examined as a potential therapeutic approach for cognitive rehabilitation, and future research directions are proposed to advance this field.

## Graphical abstract

This graphical abstract illustrates the Thyroid‐Brain‐Cognition model, summarizing the multi‐level mechanisms linking thyroid dysfunction to cognitive impairment, and emphasizes neurofeedback as a non‐invasive intervention targeting brain network modulation and neuroplasticity enhancement.

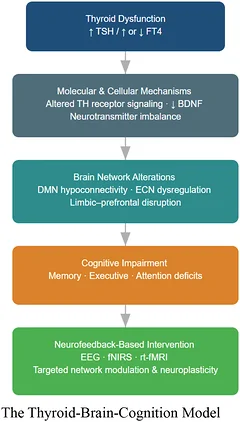

## 1 Introduction

Thyroid disorders represent a growing global health burden, with implications extending far beyond metabolic and cardiovascular complications. Recent epidemiological data highlight the increasing prevalence of thyroid dysfunction: the global age‐standardized incidence rate of thyroid cancer reached 2.914% in 2021, while in the United States, the age‐standardized prevalence of clinical thyroid dysfunction (hyperthyroidism and hypothyroidism) increased to 5.05% between 2015 and 2018, a 34.7% rise compared to 1999–2002 (Zhou et al. 2024; Zhang et al. 2023). This burden disproportionately affects older adults, with clinical hypothyroidism prevalence ranging from 0.2% to 5.7% and subclinical hypothyroidism rising sharply to 1.5%–12.5% among individuals over 65 years (Leng and Razvi 2019).

Disruptions in thyroid hormone homeostasis are associated not only with metabolic, cardiovascular, and reproductive disorders (Teixeira et al. 2020; Jabbar et al. 2017; Krassas 2000), but also with significant neuropsychiatric consequences. Emerging evidence indicates that hyperthyroidism increases the risk of attention deficits and working memory impairments (Yudiarto et al. 2006), while hypothyroidism is linked to an 81% higher incidence of Alzheimer's disease. Alarmingly, patients undergoing thyroid hormone replacement therapy face a 3.3‐fold increased risk of dementia (Wieland et al. 2022). These findings underscore the urgent need for interdisciplinary research into thyroid‐associated cognitive impairment (TACI).

Despite established associations between thyroid dysfunction and cognitive decline (Lazarus 2012), the field faces three major limitations: (1) Fragmented research scope: existing literature often focuses on isolated disease subtypes (e.g., Hashimoto's thyroiditis) or specific cognitive domains (e.g., executive function), failing to integrate multi‐level mechanisms such as molecular pathways, neuroimaging correlates, and neuroregulatory dynamics. (2) Absence of theoretical models: no existing theoretical model adequately explains how thyroid hormones dynamically modulate key brain networks (e.g., default mode network [DMN] hypoconnectivity in hypothyroidism), which hinders the development of targeted therapeutic strategies. (3) Lack of innovative interventions: novel neuroplasticity‐based approaches are largely absent from current TACI treatment frameworks, creating a gap between mechanistic understanding and clinical implementation. This gap persists despite accelerating advances in neuroendocrinology, leaving TACI without evidence‐based neurocognitive rehabilitation strategies.

We first conduct a preliminary mapping of cognitive phenotypes across euthyroid, hyperthyroid, and hypothyroid states, identifying associated neural mechanisms such as hippocampal volume reduction and prefrontal cortex dysregulation. Subsequently, we integrate multimodal neuroimaging evidence to elucidate how thyroid hormones influence brain network remodeling. Specifically, we identify the decoupling of the default mode network (DMN) in memory deficits and the disruption of the executive control network (ECN) in attentional impairments. Finally, we propose a novel translational roadmap for neurofeedback‐based interventions. Utilizing real‐time neuroimaging techniques, we aim to modulate abnormal brain network activity and harness neuroplasticity for cognitive rehabilitation—a strategy that has not been previously explored in the context of TACI.

This study introduces the first comprehensive “Thyroid–Brain–Cognition” triad model (see Figure 1), identifying DMN/ECN connectivity patterns as potential early biomarkers and validating the efficacy of neurofeedback‐driven circuit remodeling. By integrating molecular mechanisms, neuroimaging biomarkers, and advanced neuromodulation techniques, we present a potential intervention framework that transitions from reactive hormone replacement to proactive cognitive restoration. Our findings pave the way for a transformative approach to reducing the neurocognitive burden of thyroid disorders, particularly among aging populations globally.

**FIGURE 1:**
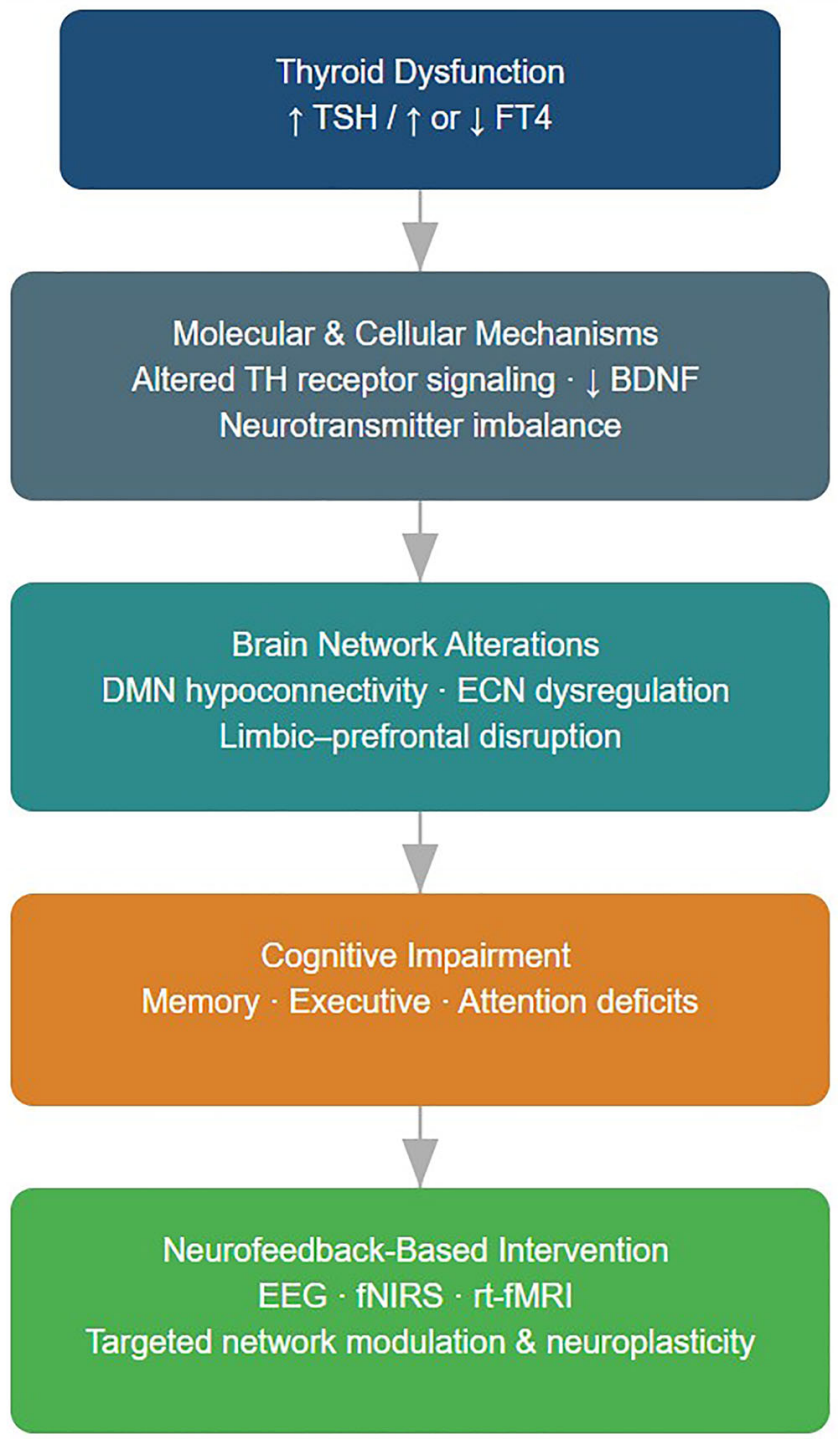
The Thyroid–Brain–Cognition model.

## 2 Methods

This systematic review was retrospectively registered in Prospero (ID: 1302279) and conducted in accordance with the PRISMA 2020 reporting guidelines. Predefined eligibility criteria, a dual‐stage screening procedure, and an auditable decision trail, including reasons for exclusion at each stage, were rigorously implemented to ensure methodological rigor and reproducibility. A comprehensive search strategy was implemented across five major electronic databases: PubMed, Embase, Scopus, Cochrane Library, and Google Scholar. The search spanned literature published from January 2000 to December 2025. The search syntax was structured around four conceptual domains, combined using Boolean operators: (1) thyroid‐cognitive interactions: (“Thyroid function AND cognitive function”); (2) hyperthyroidism‐related cognitive impairments: (“Hyperthyroidism AND cognitive impairment”); (3) hypothyroidism‐related memory disorders: (“Hypothyroidism AND cognitive impairment”); (4) intervention approaches: (“neurorehabilitation”). The initial search retrieved a total of 2497 non‐duplicate records. The selection process was documented using a PRISMA flowchart to ensure transparency and reproducibility.

A three‐phase screening protocol was applied, with dual independent reviewers involved to minimize selection bias. (1) Phase 1: Deduplication and Language Filtering: Automated tools were used to remove duplicate entries and limit results to English‐language publications, eliminating 638 records. (2) Phase 2: Title and Abstract Screening: Titles and abstracts were evaluated against predefined methodological criteria. Studies such as animal models, conference abstracts, narrative reviews, and non‐primary research articles were excluded. (3) Phase 3: Full‐Text Evaluation: A total of 232 full‐text articles underwent critical appraisal using structured eligibility criteria: (a) human participants aged ≥18 years with confirmed euthyroid, hyperthyroid, or hypothyroid status; (b) use of validated cognitive assessment tools (e.g., MoCA, MMSE) or neuroimaging biomarkers; and (c) for interventional studies, inclusion of randomized controlled trials with active control arms. Exclusion criteria included case reports; underpowered cross‐sectional studies (*n* < 20); cohorts without baseline thyroid function data; and populations with comorbid neurological conditions (e.g., stroke, neurodegenerative disorders). Discrepancies between reviewers were resolved through consensus meetings or by consulting a third reviewer. Ultimately, 44 studies met all inclusion criteria and were included in the final analysis (see Figure 2).

**FIGURE 2:**
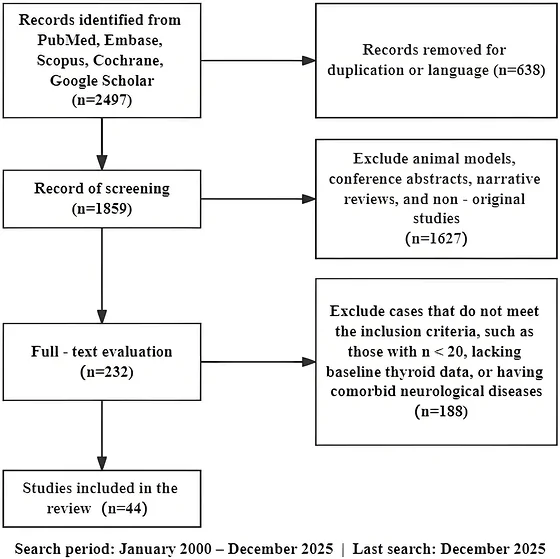
PRISMA flowchart of literature search and selection process.

## 3 Results

### 3.1 Research Framework and Synthesis Strategy

This review utilizes the PRISMA framework to synthesize current evidence on thyroid‐cognitive interactions across three clinically relevant dimensions: (1) cognitive correlates within the laboratory‐defined euthyroid range; (2) cognitive consequences of overt thyroid dysfunction; and (3) therapeutic efficacy of both conventional and emerging interventions. Systematic database searches yielded an initial pool of 2497 records. After a three‐stage screening process, 44 studies fulfilled the predefined inclusion criteria (32 observational studies; 12 interventional trials). This stratified approach enables a focused analysis of the pathophysiological continuum, ranging from subclinical hormone variations to overt dysfunction, while simultaneously identifying key gaps in the translation of mechanistic insights into neural pathways.

### 3.2 Thyroid‐Cognitive Interplay: Mechanistic and Clinical Evidence

#### 3.2.1 Euthyroid Spectrum and Neurocognitive Vulnerability

Thyroid hormones (THs) play a pivotal role in neurodevelopment, neuroplasticity, and neurotransmission, primarily regulated through the hypothalamic–pituitary–thyroid (HPT) axis (Brent 2012; Ortiga‐Carvalho et al. 2016). However, accumulating evidence indicates complex and often inconsistent associations between subtle variations in thyroid function within the clinically normal range and cognitive performance.

For instance, a two‐sample Mendelian randomization study in individuals of European ancestry found that genetically predicted higher thyrotropin (TSH) levels within the euthyroid range were associated with reduced cognitive ability and prolonged reaction times (Yu and Shan 2024). In contrast, data from the prospective Rotterdam Study (*n* = 9446; mean age 65 years) showed that higher TSH levels within the normal range were independently linked to a lower risk of incident dementia, even after adjustment for cardiovascular risk factors (Chaker et al. 2016).

Beyond linear associations, the Framingham Offspring Study (*n* = 1864 cognitively normal, euthyroid participants; mean follow‐up 12.7 years; 209 incident Alzheimer's disease [AD] cases, predominantly women) revealed a U‐shaped relationship. Women with serum TSH levels in the lowest (<1.0 mIU/L) and highest (>2.1 mIU/L) tertiles had more than double the risk of developing AD compared to those in the middle tertile (Tan et al. 2008).

In contrast, a longitudinal study of 3401 euthyroid, cognitively intact individuals (with 145 incident dementia cases during follow‐up) reported no significant association between baseline TSH levels and dementia risk. However, men who later developed dementia exhibited higher baseline free thyroxine (FT4) levels (Yeap et al. 2012). Similarly, a study of 539 euthyroid US men aged ≥65 years found no correlation between baseline TSH or FT4 levels and measures of quality of life, mood, or cognitive function (Samuels et al. 2016).

#### 3.2.2 Hyperthyroidism and Cognitive Dysfunction

Hyperthyroidism, characterized by excessive thyroid hormone (TH) secretion and symptoms such as weight loss, palpitations, and autonomic dysfunction, is increasingly associated with cognitive impairments, including attention deficits, memory decline, and reduced executive function (Jurado‐Flores et al. 2022). Notably, much of the research has focused on the link between hyperthyroidism and the risk of dementia.

Using data from the Danish National Patient Registry (56,128 hyperthyroid patients vs. 224,512 matched controls) and the OPENTHYRO Registry (2688 hyperthyroid patients vs. 10,752 matched controls), a combined analysis revealed a significantly higher incidence of all‐cause dementia among hyperthyroid patients (Danish cohort: 4.9% vs. 3.6%; OPENTHYRO cohort: 7.1% vs. 4.4%, not statistically significant). Crucially, the pooled analysis demonstrated a 16% increased relative risk of all‐cause dementia for every 6‐month reduction in TSH duration, indicating a duration‐dependent elevation in dementia risk (Folkestad et al. 2020).

Supporting these findings, the Atherosclerosis Risk in Communities Neurocognitive Study followed 12,481 participants (middle‐aged to elderly) for approximately 22 years. Although the majority had normal thyroid function, 3.4% exhibited overt hyperthyroidism (primarily exogenous). Compared with euthyroid controls, individuals with overt hyperthyroidism had a 40% higher risk of incident dementia (George et al. 2019). Collectively, these studies confirm a significant positive association between hyperthyroidism—particularly when prolonged—and an increased risk of dementia.

Recently, a limited number of interventional studies have examined the effects of normalizing thyroid function. For instance, one month of antithyroid drug treatment (e.g., methimazole) partially restored functional connectivity between the prefrontal cortex and the posterior cingulate gyrus, suggesting that some neurocognitive impairments may be reversible with early intervention (Liu et al. 2017). However, several critical knowledge gaps remain: (1) the precise neural targets of therapeutic intervention are still undefined, which hinders the development of targeted therapies; (2) whether cognitive improvement can reciprocally facilitate thyroid function recovery remains unknown; and (3) there is a lack of comparative effectiveness data across different treatment modalities.

#### 3.2.3 Hypothyroidism and Cognitive Dysfunction

Hypothyroidism, defined by insufficient thyroid hormone (TH) secretion and systemic manifestations such as fatigue, weight gain, and depression, is associated with inconsistent findings regarding domain‐specific cognitive deficits, particularly in working memory and executive function (Samuels 2014).

A study comparing 14 untreated hypothyroid patients, 10 individuals with subjective cognitive complaints, and 10 healthy controls using standardized neuropsychological assessments found significant group differences only in verbal working memory (California Verbal Learning Test), with no differences observed in attention or non‐verbal tasks (Miller et al. 2007). Consistently, data from both the Danish National Patient Registry (111,565 hypothyroid patients vs. 446,260 matched controls) and the OPENTHYRO cohort confirmed a significantly increased risk of dementia among hypothyroid patients (Danish cohort: Risk Ratio 1.22, 95% CI 1.17–1.27). The OPENTHYRO data further showed a 12% increase in dementia risk for every 6 months of elevated TSH levels (Thvilum et al. 2021). Similarly, the Mexican Study on Aging and Dementia found that definite hypothyroidism (TSH > 4.8 mIU/L and FT4 < 13 pmol/L) was significantly associated with mild cognitive impairment as assessed by the Mini‐Mental State Examination (Juárez‐Cedillo et al. 2017).

In contrast, a meta‐analysis examining the association between hypothyroidism and dementia report inconsistent conclusions. Some studies found no significant association after adjusting for vascular comorbidities (Ye et al. 2022), while others reported elevated risk across age groups and with persistent TSH elevation.

Clinically, levothyroxine (LT4) replacement therapy indeed shows potential for partial cognitive recovery. For example, 6 months of LT4 treatment in subclinical hypothyroidism restored dorsolateral prefrontal cortex (DLPFC) activation and improved performance on the *n*‐back working memory task (Zhu et al. 2006). However, systematic evaluations of treatment efficacy that prioritize neural outcomes over biochemical normalization remain limited. Notably, residual cognitive impairments often persist even after biochemical euthyroidism is achieved, suggesting the possible involvement of non‐thyroid hormone‐related mechanisms (Quinque et al. 2014). The integration of neural biomarkers into clinical decision‐making remains limited, highlighting a significant translational gap.

### 3.3 Summary of Previous Studies

Current evidence indicates complex associations between thyroid dysfunction and cognitive impairment. However, accumulating research suggests that thyroid dysfunction, including both hyperthyroidism and hypothyroidism, is associated with an elevated risk of dementia. A key observation is the duration‐dependent nature of this association, whereby longer durations of thyroid dysfunction correlate with higher rates of dementia incidence.

Nevertheless, a major limitation of prior studies is their primary focus on cognitive outcomes without directly examining the underlying neural deficits. As a result, the dynamic mechanisms by which thyroid hormone (TH) fluctuations affect neural network plasticity and contribute to cognitive decline remain poorly understood. This knowledge gap hinders the clinical differentiation between reversible functional impairments and irreversible structural damage, thereby limiting opportunities for timely and effective therapeutic intervention.

Preliminary evidence indicates that short‐term antithyroid drug treatment may partially reverse cognitive deficits in hyperthyroidism, whereas persistent impairments observed in treated hypothyroidism suggest the involvement of non‐thyroid hormone‐related mechanisms. The emergence of novel interventions, such as neurofeedback, offers promising avenues for cognitive rehabilitation in individuals with thyroid disorders. Therefore, the following sections will review the neural basis of thyroid‐associated cognitive deficits (Section 3.4) and evaluate the potential of neurofeedback as an intervention strategy (Section 3.5).

### 3.4 Neurological Mechanisms Underlying Thyroid Dysfunction and Cognitive Deficits

Recently, resting‐state functional magnetic resonance imaging (rs‐fMRI) studies have revealed widespread functional connectivity (FC) abnormalities in hyperthyroidism. Patients with acute thyrotoxic myopathy exhibit significantly enhanced FC within the sensorimotor network and the left frontoparietal network—particularly involving regions such as the right superior temporal gyrus and the anterior/posterior cerebellar lobes—alongside reduced FC in areas including the occipital lobe and angular gyrus (Li et al. 2022). Furthermore, hyperthyroid patients demonstrate functional disconnection between the default mode network (DMN) and the salience network (SN), characterized by aberrant connectivity between the posterior cingulate cortex and anterior insula (Liu et al. 2017). These findings suggest that excess thyroid hormone (TH) may disrupt the dynamic balance of large‐scale brain networks essential for cognitive function.

Abnormal hemodynamic responses in task‐relevant brain regions have also been implicated (Göbel et al. 2017). For instance, fMRI studies in both clinical and subclinical hypothyroidism consistently show reduced activation in the DLPFC and aberrant hippocampal connectivity during memory‐related tasks (Wheeler et al. 2012; Yin et al. 2013). Crucially, the strength of DLPFC‐hippocampal FC positively correlates with TH levels, and its disruption is hypothesized to underlie working memory deficits. TH deficiency appears to impair hippocampal neurogenesis and prefrontal synaptic plasticity, potentially through downregulation of brain‐derived neurotrophic factor (BDNF) and altered glutamate receptor expression (Vasilopoulou et al. 2016; Wheeler et al. 2015).

Structural neuroimaging findings corroborate these functional alterations. MRI studies report reduced hippocampal volume and decreased prefrontal cortical gray matter density in hypothyroid patients (Cooke et al. 2014), consistent with observations of apoptosis in TH‐dependent memory circuits in rodent models. Additionally, THs modulate acetylcholinesterase (AChE) activity, specifically inhibiting the G1 (cytoplasmic) and G4 (membrane‐bound) AChE isoforms in the hippocampus and striatum (Vasilopoulou et al. 2016), which may disrupt cholinergic neurotransmission critical for learning and memory.

Synthesizing current evidence (see Table 1), thyroid dysfunction appears to adversely affect three core neurocognitive systems at the network level: (1) abnormal dynamic coupling between the default mode network (DMN) and dorsal attention network (DAN), potentially contributing to attention dysregulation; (2) weakened connectivity within the limbic‐prefrontal circuitry, impairing working memory encoding and emotional regulation; and (3) reorganization of cerebellar‐cortical circuits, affecting motor learning and coordination. Despite these insights, the precise neural pathways linking TH deficiency to specific cognitive impairments, particularly in human populations, remain poorly understood. Further targeted neuroimaging investigations are warranted.

**TABLE 1:** Neuroimaging findings in hyperthyroidism and hypothyroidism.

Neuroimaging modality	Hyperthyroidism	Hypothyroidism
Rs‐fMRI	DMN–SN coupling↓ Intrinsic connectivity ↑ in sensorimotor and frontoparietal networks	DLPFC–hippocampus connectivity↓ DMN low connectivity
fMRI	Abnormal perfusion and excessive task response	DLPFC and hippocampus activation ↓ during memory task
SMRI	Limited evidence of severe atrophy	• Hippocampal volume ↓ • Prefrontal gray matter density ↓
Key affected networks/regions	DMN, SN, sensorimotor, and frontoparietal networks	Prefrontal‐limbic circuit (e.g., DLPFC, hippocampus), DMN
Associated cognitive domains	Attention, executive function	Working memory, processing speed

DMN = default mode network; SN = salience network; DLPFC = dorsolateral prefrontal cortex; Rs‐fMRI = resting‐state functional magnetic resonance imaging; fMRI = functional magnetic resonance imaging; SMRI = structural magnetic resonance imaging.

### 3.5 Neurofeedback Techniques: Potential Application for Thyroid‐Associated Cognitive Deficits

Existing literature on interventions for thyroid‐associated cognitive dysfunction remains limited, primarily focusing on cognitive outcomes as secondary effects of standard thyroid treatment (Liu et al. 2017; Zhu et al. 2006). However, evidence indicating the involvement of non‐thyroid hormone mechanisms in persistent cognitive deficits (Quinque et al. 2014) highlights the potential value of adjunctive strategies that directly target neural function to improve patient prognosis and quality of life.

Neurofeedback (NF), a non‐invasive intervention grounded in principles of neuroplasticity, enables closed‐loop modulation of specific brain regions through real‐time feedback of electroencephalogram (EEG) or hemodynamic activity (Thibault et al. 2016). It shows promise in the management of neurodegenerative conditions (Subramanian et al. 2016; Hohenfeld et al. 2017). For example, EEG‐based neurofeedback targeting the sensorimotor rhythm (SMR, 12–15 Hz) has significantly improved Montreal Cognitive Assessment (MoCA) scores in patients with mild cognitive impairment, potentially by enhancing thalamocortical inhibitory function (Marlats et al. 2020). Notably, the characteristic increase in theta band (4–7 Hz) power observed in hypothyroidism aligns with the frequency‐specific effects of SMR training, suggesting that EEG‐NF may offer a mechanism‐targeted approach for addressing thyroid‐related cognitive impairments (Wei et al. 2017).

Personalized NF protocols demonstrate sustained efficacy. Interventions tailored to individual upper alpha frequency boundaries have shown lasting memory improvements for at least 1‐month post‐intervention in randomized controlled trials (Lavy et al. 2021). These advancements underscore the critical need to identify neural biomarkers specific to different thyroid disorders, such as aberrant functional connectivity patterns and abnormal spectral power signatures, to accurately localize dysfunctional brain circuits and optimize individualized NF parameters, including target region, frequency band, and reinforcement threshold (Molcho et al. 2022).

Functional near‐infrared spectroscopy (fNIRS)‐based NF, which offers portability and moderate spatial resolution, holds considerable potential for cognitive rehabilitation. Research confirms that fNIRS‐NF targeting the left DLPFC significantly increases oxygenated hemoglobin concentration in patients with mild cognitive impairment, with neural changes strongly correlated with improvements in working memory (Lee et al. 2023). Similarly, a systematic review of 33 real‐time fMRI (rt‐fMRI)‐NF studies indicates that rt‐fMRI‐NF can enable learned modulation of specific brain signals, accompanied by measurable neural and behavioral changes (Wang et al. 2018). Collectively, these findings position neurofeedback technologies as a promising frontier for developing targeted interventions for thyroid‐associated cognitive impairments.

Although NF holds considerable therapeutic promise, its current clinical implementation faces several potential limitations. First, accessibility and cost pose substantial barriers, particularly for real‐time fMRI‐based NF, which demands highly specialized imaging infrastructure, technical expertise, and sustained operational resources. Second, interindividual variability in treatment response is pronounced; this heterogeneity likely stems from differences in disease duration, residual neural plasticity, baseline cognitive function, and the presence or severity of comorbid affective symptoms. Third, a lack of standardized protocols severely impedes progress, existing studies diverge markedly in target brain regions, electrophysiological frequency bands, session dosage and total training duration, and selection of cognitive or neurophysiological outcome measures. Fourth, the majority of published NF trials in thyroid‐related cognitive impairment remain underpowered, with small sample sizes and limited follow‐up intervals, raising concerns regarding both long‐term clinical efficacy and population‐level generalizability. Collectively, these challenges underscore the urgent need for large‐scale, multicenter randomized controlled trials and the development of biomarker‐informed, disease‐specific neurofeedback protocols tailored to thyroid dysfunction. Currently, the evidence base for neurofeedback in thyroid disorders remains limited and largely exploratory; thus, clinical translation demands rigorous validation through adequately powered, prospective, biomarker‐stratified trials.

## 4 Limitations

A key limitation of this review is its retrospective registration with PROSPERO. To uphold methodological rigor and reporting transparency, future systematic reviews should register prospectively in accordance with established guidelines.

## 5 Future Research Directions

Future research should focus on three core dimensions to establish a comprehensive, multi‐level research framework for thyroid‐related cognitive impairment. The first dimension involves elucidating the underlying mechanisms and heterogeneity of these impairments. This requires deepening investigations into the neural substrates associated with thyroid‐related cognitive deficits, with particular attention to age‐ and sex‐related variations. For instance, the significantly higher hippocampal thyroid hormone receptor β (TRβ) density observed in women, along with sex‐specific associations with AD risk, characterized by a U‐shaped pattern in postmenopausal women versus a linear trend in men—underscores the need for sex‐stratified multi‐omics analyses (Tan et al. 2008).

The second dimension focuses on developing tailored interventions, particularly through the design of neurofeedback (NF) systems specifically adapted for thyroid disorders. This approach necessitates real‐time feedback modalities capable of capturing the dynamic interactions between thyroid hormone (TH) fluctuation patterns (e.g., free thyroxine [FT4] circadian rhythm, thyroid‐stimulating hormone [TSH] pulsatility) and neuroplasticity biomarkers. Machine learning algorithms can be employed to develop predictive models of treatment response, enabling optimization of NF spatiotemporal parameters—such as training intervals aligned with TSH dynamics and region‐specific targets tailored to individual cognitive deficit profiles—and enhancing integration with levothyroxine (LT4) therapy.

Furthermore, it is essential to consider the downstream cascading effects of cognitive impairment. The long‐term consequences of thyroid‐associated cognitive dysfunction on patients’ social functioning and neurodegenerative outcomes should be systematically evaluated. This could be achieved through the development of a multidimensional predictive model that integrates neuropsychological assessments, multiple biomarker clusters, and digital phenotypic monitoring. Such a model would allow quantification of how executive function deficits contribute to declines in daily living abilities. Particular emphasis should be placed on incorporating thyroid function fluctuation parameters into predictive models of progression from mild cognitive impairment to dementia, as well as on assessing the synergistic amplification effect of combined cognitive‐motor deficits on fall risk.

Coordinated advancement across these research directions will facilitate progress along the entire translational spectrum, from basic mechanisms to clinical applications, thereby providing a robust scientific foundation for the development of precise prevention and treatment strategies for thyroid‐related cognitive impairment.

## 6 Conclusion

This review synthesizes current evidence demonstrating a complex, duration‐dependent association between thyroid dysfunction and cognitive impairment, particularly in episodic memory, working memory, and executive control, and identifies large‐scale brain network reorganization as a key neurobiological substrate. Empirical findings consistently indicate that both hyperthyroidism and hypothyroidism disrupt functional integrity within canonical neural circuits, resulting in deficits in domain‐specific cognition. Critically, such network‐level disturbances and associated cognitive impairments frequently persist despite restoration of euthyroid status through standard hormonal replacement therapy.

The “Thyroid–Brain–Cognition” triadic model integrates these observations into a coherent, multilevel framework: it posits that chronic endocrine dysregulation drives maladaptive neuroplastic changes in large‐scale networks, which mediate downstream cognitive decline. The model further proposes that neuromodulatory strategies, such as neurofeedback, represent a mechanistically grounded, adjunctive approach to conventional endocrine management. Nevertheless, the existing evidence base for neurofeedback‐based interventions remains limited and largely hypothesis‐generating. Robust clinical implementation therefore hinges on validation through adequately powered, prospective, and protocol‐standardized trials. To advance precision cognitive rehabilitation in thyroid disorders, future research should prioritize longitudinal, multimodal studies that integrate dynamic thyroid hormone profiling, quantitative neuroimaging biomarkers, and standardized cognitive phenotyping, enabling patient stratification and hypothesis‐driven intervention development.

## Author Contributions

**Mengyang Wang**: Conceptualization, methodology, investigation, data curation, writing – original draft. **Yutong Liu**: Investigation, data curation, writing – review & editing. **Mengyuan Chen**: Investigation, visualization, writing – review & editing. **Zhiqiang Ma**: Conceptualization, supervision, writing – review & editing. **Tao Liu**: Conceptualization, supervision, project administration, writing – review & editing. All authors have read and approved the final manuscript.

## Funding

This work was supported by the Henan Medical Science and Technology Research Program [Grant Number: LHGJ20200544] and the Wu Jieping Medical Foundation [Grant Number: 320.6750.2025‐21‐1].

## Ethics Statement

The authors have nothing to report.

## Data Availability

All data supporting this review are from published articles cited in the manuscript

## References

[brb371817-bib-0001] Brent, G. A. 2012. “Mechanisms of Thyroid Hormone Action.” Journal of Clinical Investigation 122, no. 9: 3035–3043. 10.1172/JCI60047. PMC3433956 22945636

[brb371817-bib-0002] Chaker, L. , F. J. Wolters , and D. Bos , et al. 2016. “Thyroid Function and the Risk of Dementia: The Rotterdam Study.” Neurology 87, no. 16: 1688–1695. 10.1212/WNL.0000000000003227. 27638924

[brb371817-bib-0003] Cooke, G. E. , S. Mullally , N. Correia , S. M. O'Mara , and J. Gibney . 2014. “Hippocampal Volume Is Decreased in Adults With Hypothyroidism.” Thyroid 24, no. 3: 433–440. 10.1089/thy.2013.0058. 24205791

[brb371817-bib-0004] Folkestad, L. , F. Brandt , M. Lillevang‐Johansen , T. H. Brix , and L. Hegedüs . 2020. “Graves' Disease and Toxic Nodular Goiter, Aggravated by Duration of Hyperthyroidism, Are Associated With Alzheimer's and Vascular Dementia: A Registry‐Based Long‐Term Follow‐Up of Two Large Cohorts.” Thyroid 30, no. 5: 672–680. 10.1089/thy.2019.0672. 31984866

[brb371817-bib-0005] George, K. M. , P. L. Lutsey , E. Selvin , P. Palta , B. G. Windham , and A. R. Folsom . 2019. “Association Between Thyroid Dysfunction and Incident Dementia in the Atherosclerosis Risk in Communities Neurocognitive Study.” Journal of Endocrinology and Metabolism 9, no. 4: 82–89. 10.14740/jem588. PMC7223793 32411312

[brb371817-bib-0006] Göbel, A. , M. Heldmann , and A. Sartorius , et al. 2017. “Mild Thyrotoxicosis Leads to Brain Perfusion Changes: An Arterial Spin Labelling Study.” Journal of Neuroendocrinology 29, no. 1. 10.1111/jne.12446. 27859916

[brb371817-bib-0007] Hohenfeld, C. , N. Nellessen , and I. Dogan , et al. 2017. “Cognitive Improvement and Brain Changes After Real‐Time Functional MRI Neurofeedback Training in Healthy Elderly and Prodromal Alzheimer's Disease.” Frontiers in Neurology 8: 384. 10.3389/fneur.2017.00384. PMC5552678 28848488

[brb371817-bib-0008] Jabbar, A. , A. Pingitore , S. H. Pearce , A. Zaman , G. Iervasi , and S. Razvi . 2017. “Thyroid Hormones and Cardiovascular Disease.” Nature Reviews Cardiology 14, no. 1: 39–55. 10.1038/nrcardio.2016.174. 27811932

[brb371817-bib-0009] Juárez‐Cedillo, T. , L. Basurto‐Acevedo , and S. Vega‐García , et al. 2017. “Prevalence of Thyroid Dysfunction and Its Impact on Cognition in Older Mexican Adults: (SADEM study).” Journal of Endocrinological Investigation 40, no. 9: 945–952. 10.1007/s40618-017-0654-6. 28343318

[brb371817-bib-0010] Jurado‐Flores, M. , F. Warda , and A. Mooradian . 2022. “Pathophysiology and Clinical Features of Neuropsychiatric Manifestations of Thyroid Disease.” Journal of the Endocrine Society 6, no. 2: bvab194. 10.1210/jendso/bvab194. PMC8765786 35059548

[brb371817-bib-0011] Krassas, G. E. 2000. “Thyroid Disease and Female Reproduction.” Fertility and Sterility 74, no. 6: 1063–1070. 10.1016/s0015-0282(00)01589-2. 11119728

[brb371817-bib-0012] Lavy, Y. , T. Dwolatzky , Z. Kaplan , J. Guez , and D. Todder . 2021. “Mild Cognitive Impairment and Neurofeedback: A Randomized Controlled Trial.” Frontiers in Aging Neuroscience 13: 657646. 10.3389/fnagi.2021.657646. PMC8236892 34194315

[brb371817-bib-0013] Lazarus, J. H. 2012. “Thyroid Hormones and Cognitive Function.” Expert review of Endocrinology & Metabolism 7, no. 4: 365–367. 10.1586/eem.12.27. 30754164

[brb371817-bib-0014] Lee, I. , D. Kim , S. Kim , H. J. Kim , U. S. Chung , and J. J. Lee . 2023. “Cognitive Training Based on Functional Near‐Infrared Spectroscopy Neurofeedback for the Elderly With Mild Cognitive Impairment: A Preliminary Study.” Frontiers in Aging Neuroscience 15: 1168815. 10.3389/fnagi.2023.1168815. PMC10410268 37564400

[brb371817-bib-0015] Leng, O. , and S. Razvi . 2019. “Hypothyroidism in the Older Population.” Thyroid Research 12: 2. 10.1186/s13044-019-0063-3. PMC6367787 30774717

[brb371817-bib-0016] Li, Y. , M. Ling , S. Huang , et al. 2022. “The Resting‐State Brain Network Functional Connectivity Changes in Patients With Acute Thyrotoxic Myopathy Based on Independent Component Analysis.” Frontiers in Endocrinology 13: 829411. 10.3389/fendo.2022.829411. PMC8986988 35399921

[brb371817-bib-0017] Liu, B. , Q. Ran , D. Liu , S. Zhang , and D. Zhang . 2017. “Changes in Resting‐State Cerebral Activity in Patients With Hyperthyroidism: A Short‐Term Follow‐Up Functional MR Imaging Study.” Scientific Reports 7, no. 1: 10627. 10.1038/s41598-017-10747-7. PMC5587688 28878279

[brb371817-bib-0018] Marlats, F. , G. Bao , and S. Chevallier , et al. 2020. “SMR/Theta Neurofeedback Training Improves Cognitive Performance and EEG Activity in Elderly With Mild Cognitive Impairment: A Pilot Study.” Frontiers in Aging Neuroscience 12: 147. 10.3389/fnagi.2020.00147. PMC7308493 32612522

[brb371817-bib-0019] Miller, K. J. , T. D. Parsons , and P. C. Whybrow , et al. 2007. “Verbal Memory Retrieval Deficits Associated With Untreated Hypothyroidism.” The Journal of Neuropsychiatry and Clinical Neurosciences 19, no. 2: 132–136. 10.1176/jnp.2007.19.2.132. 17431058

[brb371817-bib-0020] Molcho, L. , N. B. Maimon , N. Regev‐Plotnik , S. Rabinowicz , N. Intrator , and A. Sasson . 2022. “Single‐Channel EEG Features Reveal an Association With Cognitive Decline in Seniors Performing Auditory Cognitive Assessment.” Frontiers in Aging Neuroscience 14: 773692. 10.3389/fnagi.2022.773692.. PMC9191625 35707705

[brb371817-bib-0021] Ortiga‐Carvalho, T. M. , M. I. Chiamolera , C. C. Pazos‐Moura , and F. E. Wondisford . 2016. “Hypothalamus‐Pituitary‐Thyroid Axis.” Comprehensive Physiology 6, no. 3: 1387–1428. 10.1002/cphy.c150027. 27347897

[brb371817-bib-0022] Quinque, E. M. , S. Karger , K. Arélin , M. L. Schroeter , J. Kratzsch , and A. Villringer . 2014. “Structural and Functional MRI Study of the Brain, Cognition and Mood in Long‐Term Adequately Treated Hashimoto's Thyroiditis.” Psychoneuroendocrinology 42: 188–198. 10.1016/j.psyneuen.2014.01.015. 24636515

[brb371817-bib-0023] Samuels, M. H. 2014. “Psychiatric and Cognitive Manifestations of Hypothyroidism.” Current Opinion in Endocrinology, Diabetes & Obesity 21, no. 5: 377–383. 10.1097/MED.0000000000000089. PMC4264616 25122491

[brb371817-bib-0024] Samuels, M. H. , R. Kaimal , and A. Waring , et al. 2016. “Thyroid Function Variations Within the Reference Range Do Not Affect Quality of Life, Mood, or Cognitive Function in Community‐Dwelling Older Men.” Thyroid 26, no. 9: 1185–1194. 10.1089/thy.2016.0104. PMC5036313 27484219

[brb371817-bib-0025] Subramanian, L. , M. B. Morris , M. Brosnan , D. L. Turner , H. R. Morris , and D. E. Linden . 2016. “Functional Magnetic Resonance Imaging Neurofeedback‐Guided Motor Imagery Training and Motor Training for Parkinson's Disease: Randomized Trial.” Frontiers in behavioral neuroscience 10: 111. 10.3389/fnbeh.2016.00111. PMC4896907 27375451

[brb371817-bib-0026] Tan, Z. S. , A. Beiser , R. S. Vasan , et al. 2008. “Thyroid Function and the Risk of Alzheimer Disease: The Framingham Study.” Archives of Internal Medicine 168, no. 14: 1514. 10.1001/archinte.168.14.1514. PMC2694610 18663163

[brb371817-bib-0027] Teixeira, P. F. D. S. , P. B. Dos Santos , and C. C. Pazos‐Moura . 2020. “The Role of Thyroid Hormone in Metabolism and Metabolic Syndrome.” Therapeutic Advances in Endocrinology and Metabolism 11: 2042018820917869. 10.1177/2042018820917869. PMC7238803 32489580

[brb371817-bib-0028] Thibault, R. T. , M. Lifshitz , and A. Raz . 2016. “The Self‐Regulating Brain and Neurofeedback: Experimental Science and Clinical Promise.” Cortex; A Journal Devoted to the Study of the Nervous System and Behavior 74: 247–261. 10.1016/j.cortex.2015.10.024. 26706052

[brb371817-bib-0029] Thvilum, M. , F. Brandt , M. Lillevang‐Johansen , L. Folkestad , T. H. Brix , and L. Hegedüs . 2021. “Increased Risk of Dementia in Hypothyroidism: A Danish Nationwide Register‐Based Study.” Clinical Endocrinology 94, no. 6: 1017–1024. 10.1111/cen.14424. 33484007

[brb371817-bib-0030] Vasilopoulou, C. G. , C. Constantinou , D. Giannakopoulou , P. Giompres , and M. Margarity . 2016. “Effect of Adult Onset Hypothyroidism on Behavioral Parameters and Acetylcholinesterase Isoforms Activity in Specific Brain Regions of Male Mice.” Physiology & Behavior 164, no. Pt A: 284–291. 10.1016/j.physbeh.2016.06.016. 27317840

[brb371817-bib-0031] Wang, T. , D. Mantini , and C. R. Gillebert . 2018. “The Potential of Real‐Time fMRI Neurofeedback for Stroke Rehabilitation: A Systematic Review.” Cortex; A Journal Devoted to the Study of the Nervous System and Behavior 107: 148–165. 10.1016/j.cortex.2017.09.006. PMC6182108 28992948

[brb371817-bib-0032] Wei, T. Y. , D. W. Chang , and Y. D. Liu , et al. 2017. “Portable Wireless Neurofeedback System of EEG Alpha Rhythm Enhances Memory.” Biomedical Engineering Online 16, no. 1: 128. 10.1186/s12938-017-0418-8. PMC5684759 29132359

[brb371817-bib-0033] Wheeler, S. M. , M. P. McAndrews , E. D. Sheard , and J. Rovet . 2012. “Visuospatial Associative Memory and Hippocampal Functioning in Congenital Hypothyroidism.” Journal of the International Neuropsychological Society 18, no. 1: 49–56. 10.1017/S1355617711001378. 22114849

[brb371817-bib-0034] Wheeler, S. M. , V. C. McLelland , E. Sheard , M. P. McAndrews , and J. F. Rovet . 2015. “Hippocampal Functioning and Verbal Associative Memory in Adolescents With Congenital Hypothyroidism.” Frontiers in Endocrinology 6: 163. 10.3389/fendo.2015.00163. PMC4610202 26539162

[brb371817-bib-0035] Wieland, D. R. , J. R. Wieland , and H. Wang , et al. 2022. “Thyroid Disorders and Dementia Risk: A Nationwide Population‐Based Case‐Control Study.” Neurology 99, no. 7: e679–e687. 10.1212/WNL.0000000000200740. 35794019

[brb371817-bib-0036] Ye, Y. , Y. Wang , S. Li , J. Guo , L. Ding , and M. Liu . 2022. “Association of Hypothyroidism and the Risk of Cognitive Dysfunction: A Meta‐Analysis.” Journal of Clinical Medicine 11, no. 22: 6726. 10.3390/jcm11226726. PMC9694203 36431204

[brb371817-bib-0037] Yeap, B. B. , H. Alfonso , and S. A. Chubb , et al. 2012. “Higher Free Thyroxine Levels Predict Increased Incidence of Dementia in Older Men: The Health in Men Study.” The Journal of Clinical Endocrinology & Metabolism 97, no. 12: E2230–E2237. 10.1210/jc.2012-2108. 22977271

[brb371817-bib-0038] Yin, J. J. , L. M. Liao , and D. X. Luo , et al. 2013. “Spatial Working Memory Impairment in Subclinical Hypothyroidism: An FMRI Study.” Neuroendocrinology 97, no. 3: 260–270. 10.1159/000343201. 22986643

[brb371817-bib-0039] Yu, Z. W. , and Z. Y. Shan . 2024. “Thyroid Function Variations Within the Reference Range and Cognitive Function: A Two‐Sample Mendelian Randomization Study.” Journal of Affective Disorders 357: 156–162. 10.1016/j.jad.2024.05.007. 38703900

[brb371817-bib-0040] Yudiarto, F. L. , L. Muliadi , D. Moeljanto , and B. Hartono . 2006. “Neuropsychological Findings in Hyperthyroid Patients.” Acta Medica Indonesiana 38, no. 1: 6–10.. 16479025

[brb371817-bib-0041] Zhang, X. , X. Wang , H. Hu , H. Qu , Y. Xu , and Q. Li . 2023. “Prevalence and Trends of Thyroid Disease Among Adults, 1999‐2018.” Endocrine Practice 29, no. 11: 875–880. 10.1016/j.eprac.2023.08.006. 37619827

[brb371817-bib-0042] Zhou, T. , X. Wang , and J. Zhang , et al. 2024. “Global Burden of Thyroid Cancer From 1990 to 2021: A Systematic Analysis From the Global Burden of Disease Study 2021.” Journal of Hematology & Oncology 17, no. 1: 74. 10.1186/s13045-024-01593-y. PMC11348565 39192360

[brb371817-bib-0043] Zhu, D. F. , Z. X. Wang , and D. R. Zhang , et al. 2006. “fMRI Revealed Neural Substrate for Reversible Working Memory Dysfunction in Subclinical Hypothyroidism.” Brain 129, no. Pt 11: 2923–2930. 10.1093/brain/awl215. 16921178

